# Supplementary material for: The effects of genital myiasis on the diversity of the vaginal microbiota in female Bactrian camels
Source: BMC Vet Res. 2022 Mar 5;18:87. doi: 10.1186/s12917-022-03189-5 (PMC8897907; doi:10.1186/s12917-022-03189-5)
Supplement: Supplementary file 5 — Additional file 5. [file 12917_2022_3189_MOESM5_ESM.zip › MPL201709200_16s_yy/Treat1/B12_pca/PCA_Treat1.pdf]

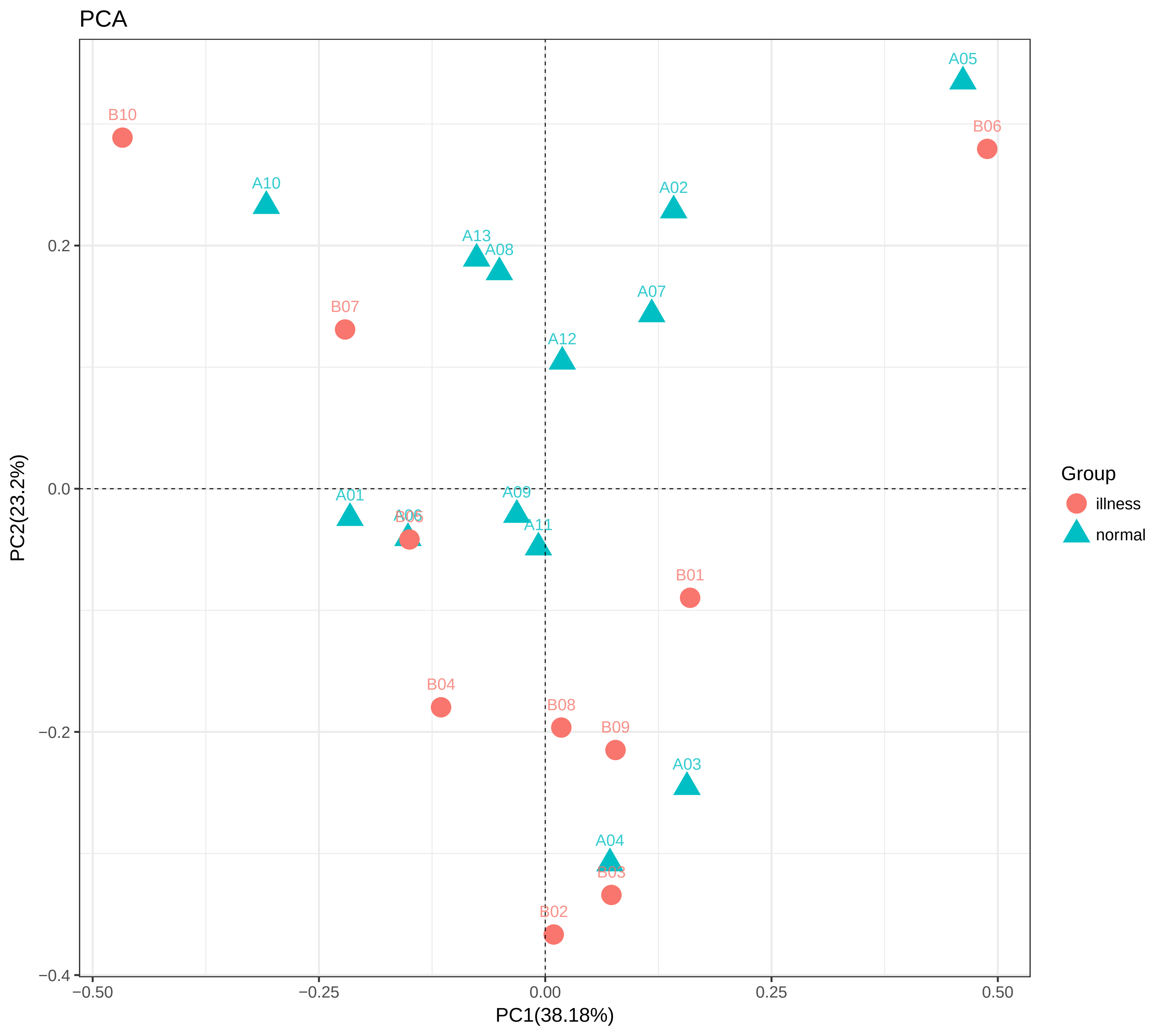
PCA
PC2(23.2%)
PC1(38.18%)
0.2
0.0
−0.2
−0.4
−0.50
−0.25
0.00
0.25
0.50
Group
illness
normal
B10
A10
B07
A13
A08
A12
A07
A02
A05
B06
A01
A09
A11
B01
B04
B08
B09
A03
A04
B02
